# Dr. Livingstone’s Fight with a Lion

**Published:** 1874-11-01

**Authors:** 


### DR. LIVINGSTONE'S FIGHT WITH A LION. A CAST OF HIS FRACTURED HUMERUS RECEIVED IN CHICAGO.

A SPECIMEN of much interest, both scientific and historical, has just been sent to the museum of the Chicago Medical College by Sir Wm. Fergusson, Baronet, the eminent surgeon of London.

The history of the case is this: Livingstone, as will be remembered, commenced his extraordinary explorations in the interior of Africa more than twenty years ago. In one of his earlier journeys he stopped for a time at a native village, whose inhabitants were much annoyed by the depredations of lions. As the killing of one or two lions usually has the effect to frighten away all the rest from the vicinity, Dr. Livingstone determined to do the timid villagers a kindness, by heading with his men a grand lion hunt to destroy part of the beasts, and intimidate the remainder. For this purpose, he and his men led the people out, and with them surrounded the lions on a wooded hill and began to contract the circle by marching the men towards the centre, but the villagers not being very courageous gave way before the charges of some of the enclosed animals, broke the circle and allowed them to escape. Finding the hunt a failure all parties returned to the village, but Livingstone and two of his men came upon one of the lions as they went, the result of which is described by himself as follows: (*Travels and Researches in South Africa*, p. 12.)

"Being about thirty yards off, I took good aim at his body, and fired both barrels into it. . . . Turning to the people I said, 'Stop a little, till I load again.' When in the act of ramming down the bullets, I heard a shout. Starting and looking half around, I saw the lion just in the act of springing upon me. I was upon a little height. He caught my shoulder as he sprang and we both came to the ground below together. Growling horribly close to my ear, he shook me as a terrier dog does a rat. The shock produced a stupor similar to that which seems to be felt by a mouse after the first shake of a cat. It caused a sort of dreaminess, in which there was no sense of pain or feeling of terror, though quite conscious of all that was happening. It was like what patients partially under the influence of chloroform describe, who see all the operation, but feel not the knife. The shake annihilated fear and allowed no sense of horror in looking round at the beast. This peculiar state is probably produced in all animals killed by the carnivora, and, if so, is a merciful provision by our Creator for lessening the pain of death.

"Turning round to relieve myself of the weight, as he had one paw on my head, I saw his eyes directed to Mebalwe, who was trying to shoot him at a distance of ten or fifteen yards. His gun missed fire. The lion immediately left me, and, attacking Mebalwe, bit his thigh. Another man, whose life I had saved before, attempted to spear the lion while he was biting Mebalwe. He left Mebalwe, and caught this man by the shoulder; but, at that moment, the bullets he had received took effect, and he fell down dead. The whole was the work of a few moments, and must have been his paroxysms of dying rage."

The lion in shaking Livingstone seized him by the left arm, fracturing the humerus just above the middle. The result was a non-union of the fracture, producing a false joint, and an overlapping of the fragments exceeding an inch in extent. The lower fragment was also rotated on its axis about ninety degrees from its natural position.

The cast shows that either from the injury to the nutrient artery, or else from diminished use of the limb, the shaft of the upper fragment became very much atrophied.

When Livingstone's body was received in London, some natural doubt was felt about its identity, but Sir Wm. Fergusson proved by examination of the fractured bone and false joint, that there could be no uncertainty in the case. The cast is now in the museum of the Chicago Medical College.

The hunters of South Africa have observed that the bites of lions are very troublesome in their healing, and hence believe that the saliva of the animal is poisonous. Dr. Livingstone himself was half inclined to think that there was some truth in the idea, and thought that his own exemption from virulent symptoms might perhaps be due to his having been bitten through his clothing.

Some professional men in Chicago have been partially impressed in the same way from the persistent inflammation observed in a patient in one of the hospitals, who was bitten in the hand by a lion in a menagerie some months ago, and is still far from being cured. I am not aware, however, of any actual, experimental proof of poisonous qualities in the leonine saliva, and the fact that the teeth of the animal make punctured and lacerated wounds, often penetrating joints and comminuting bones, is a sufficient mechanical reason why many of the wounds should do badly.

